# Normative high-frequency oscillation phase–amplitude coupling and effective connectivity under sevoflurane

**DOI:** 10.1093/braincomms/fcaf403

**Published:** 2025-10-15

**Authors:** Ethan Firestone, Hiroshi Uda, Naoto Kuroda, Kazuki Sakakura, Masaki Sonoda, Riyo Ueda, Yu Kitazawa, Min-Hee Lee, Jeong-Won Jeong, Aimee F Luat, Michael J Cools, Sandeep Sood, Eishi Asano

**Affiliations:** Department of Pediatrics, Children’s Hospital of Michigan, Detroit Medical Center, Wayne State University, Detroit, MI 48201, USA; Department of Physiology, Wayne State University, Detroit, MI 48201, USA; Department of Pediatrics, Children’s Hospital of Michigan, Detroit Medical Center, Wayne State University, Detroit, MI 48201, USA; Department of Neurosurgery, Osaka Metropolitan University Graduate School of Medicine, Osaka 5458585, Japan; Department of Pediatrics, Children’s Hospital of Michigan, Detroit Medical Center, Wayne State University, Detroit, MI 48201, USA; Department of Epileptology, Tohoku University Graduate School of Medicine, Sendai 9808575, Japan; Department of Pediatrics, Children’s Hospital of Michigan, Detroit Medical Center, Wayne State University, Detroit, MI 48201, USA; Department of Neurosurgery, University of Tsukuba, Tsukuba, Ibaraki 3058575, Japan; Department of Pediatrics, Children’s Hospital of Michigan, Detroit Medical Center, Wayne State University, Detroit, MI 48201, USA; Department of Neurosurgery, Yokohama City University Graduate School of Medicine, Yokohama, Kanagawa 2360004, Japan; Department of Pediatrics, Children’s Hospital of Michigan, Detroit Medical Center, Wayne State University, Detroit, MI 48201, USA; Department of Neurosurgery, National Center Hospital, National Center of Neurology and Psychiatry, Tokyo 1878551, Japan; Department of Pediatrics, Children’s Hospital of Michigan, Detroit Medical Center, Wayne State University, Detroit, MI 48201, USA; Department of Neurology and Stroke Medicine, Yokohama City University, Yokohama, Kanagawa 2360004, Japan; Department of Pediatrics, Children’s Hospital of Michigan, Detroit Medical Center, Wayne State University, Detroit, MI 48201, USA; Department of Pediatrics, Children’s Hospital of Michigan, Detroit Medical Center, Wayne State University, Detroit, MI 48201, USA; Department of Neurology, Children’s Hospital of Michigan, Detroit Medical Center, Wayne State University, Detroit, MI 48201, USA; Department of Pediatrics, Children’s Hospital of Michigan, Detroit Medical Center, Wayne State University, Detroit, MI 48201, USA; Department of Neurology, Children’s Hospital of Michigan, Detroit Medical Center, Wayne State University, Detroit, MI 48201, USA; Department of Pediatrics, Central Michigan University, Mt. Pleasant, MI 48858, USA; Department of Neurosurgery, Children’s Hospital of Michigan, Detroit Medical Center, Detroit, MI 48201, USA; Department of Neurosurgery, Children’s Hospital of Michigan, Detroit Medical Center, Detroit, MI 48201, USA; Department of Pediatrics, Children’s Hospital of Michigan, Detroit Medical Center, Wayne State University, Detroit, MI 48201, USA; Department of Neurology, Children’s Hospital of Michigan, Detroit Medical Center, Wayne State University, Detroit, MI 48201, USA

**Keywords:** acute electrocorticography (ECoG), general anaesthesia, dynamic tractography, transfer-entropy-based effective connectivity, modulation-index-based phase–amplitude coupling

## Abstract

Resective surgery for paediatric drug-resistant focal epilepsy often requires extraoperative intracranial electroencephalography recording to accurately localize the epileptogenic zone. This procedure entails multiple neurosurgeries, intracranial electrode implantation and explantation, and days of invasive in-patient evaluation. There is a need for methods to reduce the diagnostic burden and introduce objective epilepsy biomarkers. Our preliminary studies aimed to address these issues by using sevoflurane anaesthesia to rapidly and reversibly activate intraoperative phase–amplitude coupling between delta and high-frequency activities, as well as high-frequency activity-based effective connectivity. Phase–amplitude coupling can serve as a proxy for spike-and-wave discharges, and effective connectivity describes the spatiotemporal dynamics of neural information flow among regions. Notably, sevoflurane activated these interictal electrocorticography biomarkers most robustly in areas whose resection led to seizure freedom. However, they were also increased in normative brain regions that did not require removal for seizure control. Before using these electrocorticography biomarkers prospectively to guide resection, we should understand their endogenous distribution and propagation pathways at different anaesthetic stages. In the current study, we highlighted the normative distribution of delta and high-frequency activity phase–amplitude coupling and effective connectivity under sevoflurane. Normative data were derived from 19 patients, whose ages ranged from 4 to 18 years and included 11 males. All achieved seizure control following focal resection. Electrocorticography was recorded at an isoflurane baseline, during stepwise increases in sevoflurane concentration, and also during extraoperative slow-wave sleep without anaesthesia. Normative electrode sites were then mapped onto a standard cortical surface for anatomical visualization. Dynamic tractography traced white matter pathways that connected sites with significantly augmented biomarkers. Finally, we analysed all sites—regardless of normal or abnormal status—to determine whether sevoflurane-enhanced biomarker values could intraoperatively localize the epileptogenic sites. We found that normative electrocorticography biomarkers increased as a function of sevoflurane concentration, especially in bilateral frontal and parietal lobe regions (Bonferroni-corrected *P*-values < 0.05). Callosal fibres directly connected homotopic Rolandic regions exhibiting elevated phase–amplitude coupling. The superior longitudinal fasciculus linked frontal and parietal association cortices, showing augmented effective connectivity. Higher biomarker values, particularly at 3–4 vol% sevoflurane, characterized epileptogenicity and seizure-onset zone status (Bonferroni-corrected *P*-values < 0.05). Supplementary analysis showed that epileptogenic sites exhibited less augmentation in delta-based effective connectivity. This study helps clarify the normative distribution of, and plausible propagation pathways supporting, sevoflurane-enhanced electrocorticographic biomarkers. Future work should confirm that sevoflurane-activated electrocorticography biomarkers can predict postoperative seizure outcomes in larger cohorts to establish their clinical utility.

## Teaser

Firestone *et al*. used interictal electrocorticography to visualize the anatomical distribution of delta (3–4 Hz) and high-frequency oscillation (80–300 Hz) phase–amplitude coupling and effective connectivity, under sevoflurane, along with plausible white matter propagation pathways. This work will improve the interpretation of these signals for the intraoperative localization of the epileptogenic zone.

## Graphical Abstract

**Graphical Abstract:**
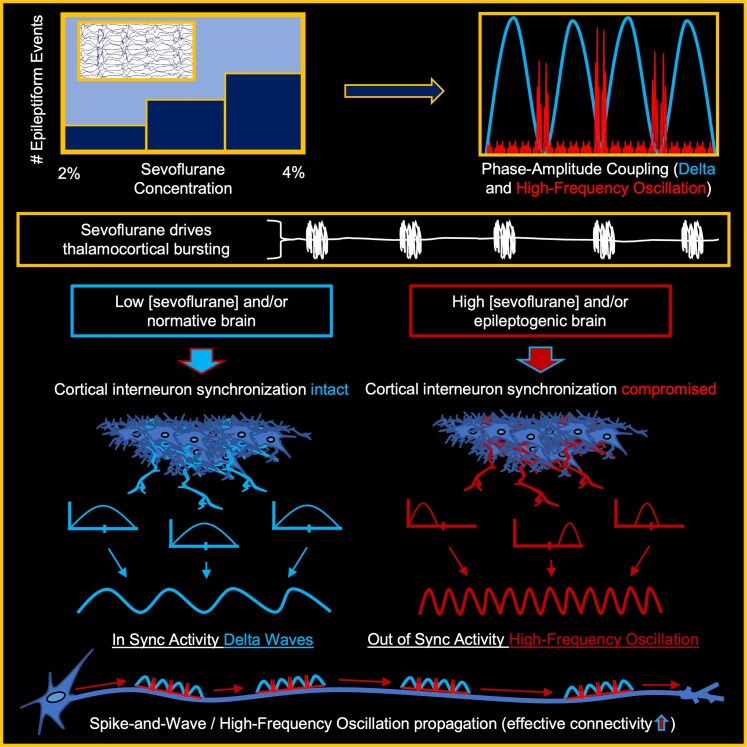

## Introduction

Millions of children are impacted by focal epilepsy, and prompt treatment is needed to avoid cognitive comorbidities.^1-3^ Around 30% of these patients will develop drug-resistant epilepsy that often requires surgery to remove the epileptogenic zone responsible for generating habitual seizures.^3-6^ Since localizing the epileptogenic zone using noninvasive modalities alone can be challenging, many patients require intracranial EEG (iEEG) electrode implantation and days of extraoperative iEEG monitoring to capture spontaneous ictal events and identify the origin of these discharges.^7-15^ The two-stage procedure involves significant risk factors from multiple neurosurgeries and chronic brain implants.^11,16,17^ It is thus ideal to acutely record intraoperative electrocorticography (ECoG) directly from the cortical surface, immediately followed by focal resection.^18,19^ Moreover, conventional methods rely on visual inspection of iEEG traces to identify epileptiform signals, such as spike-and-wave discharges. This is subject to inter-rater variability and makes it difficult to compare relative levels of epileptogenicity across sites.^20,21^ Hence, better intraoperative techniques using inducible, objective ECoG measures are needed to avoid these pitfalls.

Sevoflurane may offer a unique solution because it rapidly and reversibly augments interictal epileptiform activity, unlike other general anaesthetics that suppress such signals.^22-39^ Furthermore, recent studies have shown that sevoflurane enhances objective ECoG epilepsy biomarkers, including the occurrence rates of high-frequency oscillations (HFOs) at ≥80 Hz, phase–amplitude coupling between the 3 to 4 Hz delta phase and HFO amplitude (delta–HFO phase–amplitude coupling, delta–HFO PAC), and HFO-based effective connectivity (EC), particularly in epileptogenic regions.^40-72^ Delta–HFO PAC is considered a reliable proxy for interictal spike-and-wave discharges.^57-59,62^ Thus, we expected that sites with elevated values might serve as key nodes within networks where epileptiform signals originate and propagate. We also expected that HFO-based EC might characterize the spatiotemporal dynamics of high-frequency activity propagation within such networks.^69-71^ However, our recent investigations demonstrated that sevoflurane, likewise, increased delta–HFO PAC and HFO-based EC in normative brain regions that need not be resected for seizure freedom.^68,69^ These results highlight the need to determine the endogenous distribution of ECoG biomarkers across different sevoflurane levels. Additionally, identifying the structural pathways that mediate sevoflurane-induced signal propagation is valuable. We believe that characterizing the expected response in the normal brain will facilitate the use of sevoflurane anaesthesia for intraoperative localization of the epileptogenic zone.

As such, we analysed a limited paediatric ECoG dataset to begin deducing the normative distribution of interictal delta–HFO PAC (quantified by the modulation index^62-66^) and HFO-based EC (quantified by the transfer entropy^70,71^), at various anaesthetic stages. We next employed dynamic tractography to trace major white matter pathways that directly connected cortical sites showing significant ECoG biomarker augmentation. Finally, we determined the fidelity of using our objective, intraoperative ECoG biomarkers for characterizing epileptogenicity and seizure-onset zone (SOZ) status under sevoflurane. We hypothesized that sevoflurane would significantly augment delta–HFO phase amplitude coupling and HFO EC in normative electrode sites. We further postulated that cortical sites showing the greatest degree of co-augmentation would be linked by underlying white matter fibres. We finally hypothesized that sevoflurane would elevate these objective, intraoperative ECoG biomarkers most in epileptogenic and SOZ sites.

## Materials and methods

### General methods

We investigated intraoperative, interictal ECoG data from 19 paediatric, drug-resistant focal epilepsy patients who underwent resective surgery at the Children’s Hospital of Michigan (Detroit, MI, USA) and achieved International League Against Epilepsy (ILAE) Class I seizure outcomes. This study was strictly observational; all data were collected during standard-of-care treatment for drug-resistant focal epilepsy, with no deviation for scientific curiosity. The results were not available to influence surgical plans. Two-stage patients (*n* = 11) went through the following clinical course: (i) non-invasive presurgical evaluation^9^; (ii) implantation of intracranial electrodes in conjunction with sevoflurane-activated, intraoperative ECoG; (iii) 3–5 days of extraoperative functional brain mapping via iEEG recording; (iv) a second neurosurgery for explantation of intracranial electrodes immediately followed by focal cortical resection; and (v) 1-year postoperative follow-up to determine seizure outcome status. The study also included single-stage (*n* = 8) patients who received: (i) non-invasive presurgical evaluation; (ii) acute, intraoperative ECoG recording, immediately followed by focal cortical resection; and (iii) 1-year postoperative follow-up to determine seizure status. The statistical models addressed potential bias between one- versus two-stage surgeries. Importantly, all 19 patients experienced intraoperative ECoG recording in the presence of stepwise-rising sevoflurane. This procedure is standard of care at the Children’s Hospital of Michigan to optimize intracranial electrode sampling of implicated brain regions for invasive, extraoperative iEEG recording.

We retrospectively employed signal processing on the intraoperative ECoG and extraoperative iEEG data. We calculated delta–HFO PAC (rated by modulation index) and HFO EC (rated by transfer entropy) under stepwise increasing sevoflurane (2–4 vol%), isoflurane (1 vol%) and during slow-wave sleep (SWS); the latter two conditions were redundant controls. A linear mixed model tested whether normative delta–HFO PAC and/or HFO EC increased as a function of sevoflurane concentration. Normative electrode sites were defined as those outside the resection area, the SOZ,^9^ areas generating interictal epileptiform discharges,^61^ and regions with MRI-visible lesions.^73,74^ Pooled, normative electrodes were then interpolated onto a standard cortical template to visualize the anatomical distribution of delta–HFO PAC and HFO EC, at each anaesthetic stage. Diffusion-weighted imaging (DWI) delineated white matter streamlines connecting site-pairs with co-augmented ECoG biomarkers, in ‘dynamic tractography’.^66^ Next, we included all electrode sites, regardless of normality, to deduce whether relatively greater sevoflurane-induced augmentation of delta–HFO PAC and/or HFO EC could determine the epileptogenic status of electrodes. The epileptogenic zone was defined as the resected cortical area in patients who achieved 1-year postoperative seizure freedom.^69^ Supplementary analysis tested whether the biomarkers could likewise characterize the SOZ^9^: the area of ictogenesis within the epileptogenic zone that is prospectively defined by board-certified clinical neurophysiologists. Since spike-and-wave discharges characteristically contain a 3–4 Hz component, and delta waves are the other variable in PAC,^57,59,61,62^ we also created a supplementary normative distribution, along with prediction models, for 3–4 Hz delta EC (rated by transfer entropy).

A final supplementary analysis tested whether more complete resection of high-value ECoG biomarker sites could predict ILAE seizure outcomes. This analysis included an extra four patients (aged 6–20 years; 3 males) who underwent two-stage surgery, including sevoflurane-based ECoG and extraoperative iEEG recordings, but failed to achieve ILAE Class I seizure outcomes. Their detailed clinical information can be found in Supplementary Table 1.

### Patient population

The study included 19 paediatric patients (aged 4–18 years; 11 males) who received resective epilepsy surgery and achieved 1-year postoperative ILAE-defined seizure freedom. We obtained written consent from all patients or the legal guardians of those under 18 years old and from those who were unable to provide their own consent. Detailed clinical characteristics can be found in Table 1. All analyses were approved by the Institutional Review Board at Wayne State University, Detroit, MI, USA.

**Table 1:** Patient profiles

Patient number	Age (years)	Biological sex	Number of anti-seizure medications	Number of electrodes total (normative)	One- versus two-stage surgery	MRI finding
1	15	M	1	32 (26)	1	Tumor
2	13	M	2	112 (67)	2	Dysplasia
3	4	M	1	60 (26)	1	Tumor
4	16	F	3	136 (90)	2	Nonlesional
5	9	M	1	110 (71)	2	Tumor
6	11	M	5	100 (45)	2	Dysplasia
7	13	F	3	132 (60)	2	Nonlesional
8	12	F	3	152 (130)	2	Dysplasia
9	10	F	0	64 (29)	1	Tumor
10	18	M	1	32 (26)	1	Tumor
11	17	F	0	20 (19)	1	Tumor
12	11	M	1	110 (63)	2	Tumor
13	17	M	3	96 (76)	2	Tumor
14	13	M	3	134 (88)	2	Nonlesional
15	13	M	0	32 (22)	1	Tumor
16	7	F	1	120 (42)	2	Tumor
17	8	F	2	142 (84)	2	Dysplasia
18	9	F	1	88 (47)	1	Tumor
19	11	M	0	32 (28)	1	Tumor

*Inclusion criteria*: (i) children, aged 4–20 years, with a diagnosis of drug-resistant focal epilepsy; (ii) underwent resective epilepsy surgery; and (iii) intraoperative ECoG was recorded under isoflurane maintenance and a short period of sevoflurane.

*Exclusion criteria*: (i) malignant brain tumour; (ii) progressive neurodegenerative or metabolic disorder; (iii) presence of a major developmental brain malformation, which can confound anatomical landmark identification; and (iv) previous epilepsy surgery.

### Implantation of intracranial electrodes and intraoperative ECoG

Intracranial electrodes were implanted as per standard clinical management of drug-resistant focal seizures.^9,62^ First, a multidisciplinary group of board-certified clinicians synthesized non-invasive neuroimaging, neurophysiology and neuropsychological data to estimate the location of the epileptogenic zone. Paediatric neurosurgeons then implanted intracranial electrodes on the implicated brain hemisphere(s) to be subsequently used for extraoperative, iEEG functional brain mapping. Anaesthesia induction—via propofol, fentanyl and rocuronium—and maintenance—via isoflurane (1 vol%)—were conducted as previously published.^72^ Patients were implanted with either surface platinum disc electrodes (10 mm centre-to-centre), depth electrodes (5 mm centre-to-centre) or a combination. The number and spatial extent of iEEG electrodes were strictly based on clinical needs, with no deviation for scientific curiosity. ECoG signals were recorded using a 1000 Hz sampling rate and a bandpass filter at 0.016–300 Hz. Anaesthesia was then switched to sevoflurane for 15 min to temporarily and reversibly augment interictal epileptiform discharges, reducing the risk of sampling errors.^72^ Sevoflurane concentration was stepwise increased from 2, to 3, and to 4 vol%. These doses are clinically acceptable.^28,32,68,75^ A board-certified clinical neurophysiologist interpreted the intraoperative ECoG in real-time and communicated findings to the neurosurgeon, who placed additional iEEG electrodes as needed.^72^

### Extraoperative iEEG and focal cortical resection

After implantation of intracranial iEEG electrodes, patients were transferred to the epilepsy monitoring unit for 3–5 days of extraoperative iEEG recording to delineate the epileptogenic zone and eloquent cortex, defined by electrical stimulation mapping.^9,62,76^ iEEG signals were recorded using a 1000 Hz sampling rate and a bandpass filter at 0.016–300 Hz. Each patient’s electrodes were co-registered to their reconstructed MRI, as previously reported.^62,77,78^ Board-certified clinical neurophysiologists used this data to determine the location of the SOZ.^9^ Focal resection was then carried out by a neurosurgical team aiming to remove the presumed epileptogenic zone (i.e. SOZ and neighbouring MRI lesions), while preserving eloquent cortex to avoid creating sensorimotor and/or cognitive deficits. In the single-stage patients, acute, intraoperative ECoG was recorded during the same stepwise sevoflurane paradigm described above to confirm resection margins. This was followed immediately by focal resection.

### Definition of anaesthetic stages

Intraoperative ECoG recordings were split into the following 5-min anaesthetic stages for analysis: (i) isoflurane 1 vol% (Iso); (ii) sevoflurane 2 vol% (Sev2); (iii) sevoflurane 3 vol% (Sev3); and (iv) sevoflurane 4 vol% (Sev4). The 11 two-stage patients also included (v) extraoperative iEEG without anaesthesia during SWS. Each anaesthetic condition was further subdivided into 1-min epochs (e.g. ‘Sev2_1’ refers to the first minute of sevoflurane 2 vol%). All anaesthetic concentrations were within clinical limits.^75^ Conditions (i) and (v) were redundant controls.

### Definition of normative, epileptogenic and seizure-onset zones

We analysed iEEG data from a total of *n* = 1608 electrode sites (isoflurane control) and *n* = 1344 (SWS control). Note, both controls involved the same electrode pool. The different electrode counts in isoflurane versus SWS reflected the different number of patients between combined one- and two-stage surgery patients (i.e. isoflurane control) versus only two-stage surgery patients (i.e. SWS control). These separate analyses were conducted to utilize redundant controls, because only the two-stage surgery patients underwent extraoperative iEEG recording without anaesthesia, during SWS. Since all patients achieved ILAE Class I seizure freedom, electrode sites that were retained in the brain, outside of the SOZ and MRI lesions, and free from interictal epileptiform discharges were retrospectively considered ‘normative’, as done previously.^66,73,74^ This included *n* = 963 normative sites (isoflurane control) and *n* = 816 normative sites (SWS control). For the prediction models, resected electrode sites in patients who achieved Class I seizure freedom were deemed ‘epileptogenic’ (isoflurane control: *n* = 475 | SWS control: *n* = 441), while those retained in the brain were ‘non-epileptogenic’ (isoflurane control: *n* = 1133 | SWS control: *n* = 903), as done previously.^68,69^ Intraoperative photographs confirmed electrode classifications, as done previously.^62^ In two-stage patients, a board-certified, clinical neurophysiologist prospectively identified areas initially exhibiting sustained rhythmic iEEG discharges, accompanied by subsequent clinical seizure activity, which were not explained by state changes; such brain areas were assigned as ‘seizure-onset zone’ (SOZ: *n* = 76).^72^ The remaining electrode sites were classified as ‘non-seizure-onset zone’ (non-SOZ: *n* = 1268).

### Electrophysiology and imaging analysis workflow

**Figure 1:**
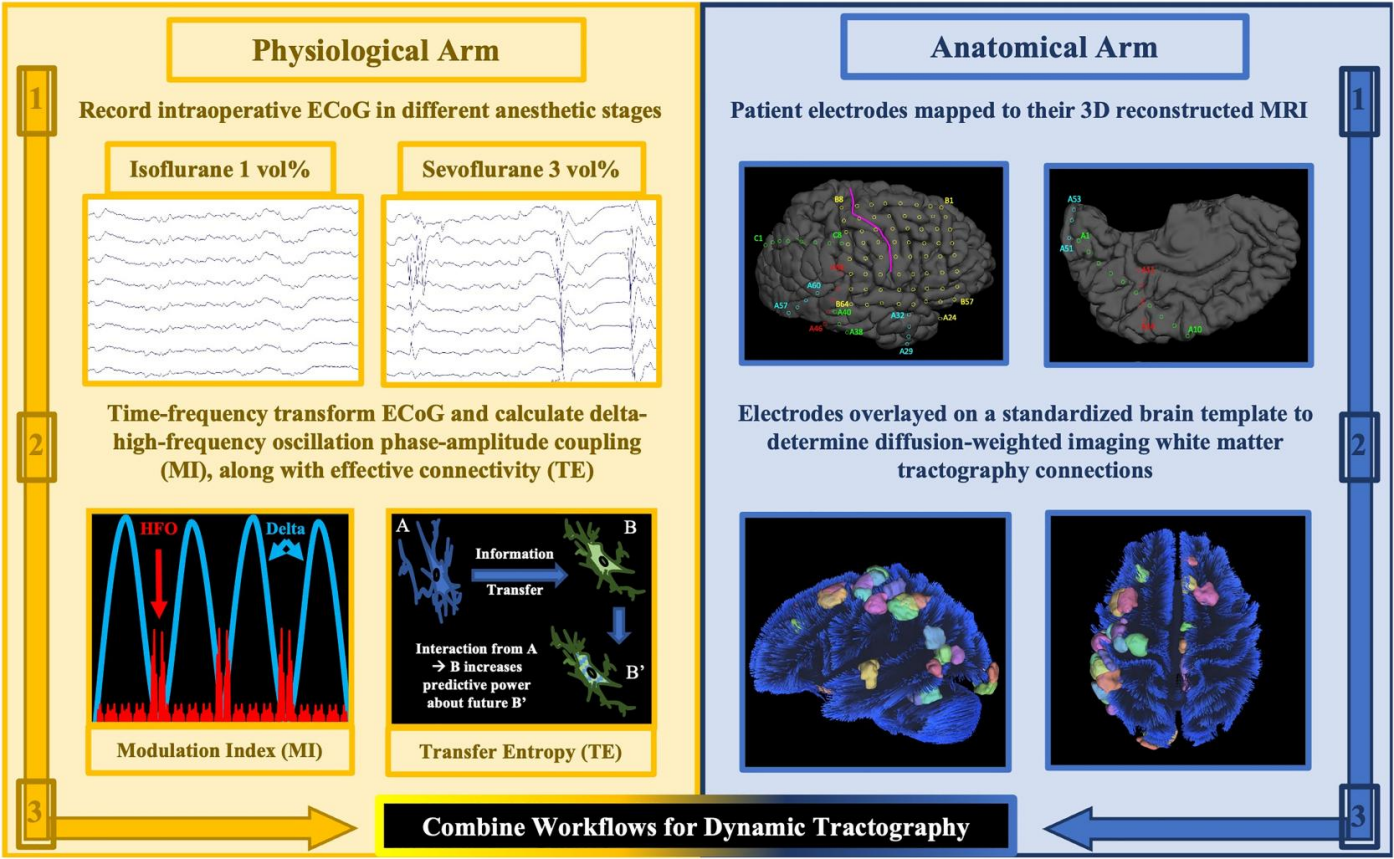
**Electrophysiology and imaging analysis workflow.** Physiological (*left*) and anatomical (*right*) arms are combined to determine the group-level distribution of normative delta (3–4 Hz) and HFO (80–300 Hz) phase–amplitude coupling (quantified by the modulation index: MI) and EC (quantified by the transfer entropy: TE). Dynamic tractography delineates white matter streamlines connecting electrophysiological cortical hotspots.

This study combined physiological and anatomical workflows, allowing for precise localization of ECoG/iEEG signals (Fig. 1). The physiological arm began with raw ECoG/iEEG recordings under various anaesthetic stages. Signals were preprocessed and time–frequency transformed to yield the spectral amplitude (square root of power) of HFO (80–300 Hz) and delta (3–4 Hz) frequency bands. These measures were then fed into modulation-index^62,68,79^ and transfer-entropy algorithms,^69-71^ calculating phase–amplitude coupling and EC, respectively. On the anatomical arm, electrode positions were interpolated onto 3D MRI scans of each individual patient’s brain. Electrodes were then spatially normalized and pooled onto a standard cortical surface and white matter templates. Joining the two workflows in dynamic tractography^66,69^ created spatially accurate cortical maps of ECoG/iEEG biomarker activity, along with white matter tracts connecting electrophysiological hotspots.

### ECoG/iEEG preprocessing and time–frequency transformation

Investigators blind to the epileptogenic status of electrodes bandpass filtered the raw ECoG/iEEG from 0.016 to 300 Hz, notch-filtered 60 Hz noise, and exported the traces onto a bipolar montage. Preprocessing and time–frequency transformation were conducted using FieldTrip (https://www.fieldtriptoolbox.org/),^80^ open-source software, via Matlab R2022b (MathWorks, Natick, MA, USA). Visual screening removed channels and recording epochs polluted with artefacts. The wavelet method calculated power spectra of HFO (80–300 Hz) and delta waves (3–4 Hz). Taking the square root of power produced a spectral amplitude time series.^69^ The spectral amplitude of each individual electrode and time point was *z*-scored based on the given channel’s mean and standard deviation during the isoflurane control period. In two-stage patients, a separate, redundant dataset was made by *z*-scoring via the SWS control.

### Calculating phase–amplitude coupling via modulation index

The modulation index was calculated, as done previously.^62,66,68,79^ The cleaned, raw bipolar ECoG/iEEG data were fed into the open-source winPACT toolbox of EEGLAB (https://sccn.ucsd.edu/wiki/WinPACT) to compute the modulation index.^79,81^ This software tool uses the Hilbert transform on an EEG time series to quantify the strength of coupling between the instantaneous phase of delta waves (3–4 Hz) and HFO (80–300 Hz) amplitude. For each channel, PAC was calculated locally to determine the degree of coupling between the 3–4 Hz delta phase and HFO amplitude at that specific site. We chose to investigate HFO coupling exclusively with 3–4 Hz delta waves, because previous studies from independent groups demonstrated that this specific narrow delta band is most representative of interictal spike-and-wave discharges.^57,58,66^

### Calculating effective connectivity via transfer entropy

Transfer entropy was calculated, as done previously.^69-71^ The *z*-scored spectral amplitude time-series data were first time-binned (each bin at least six wave-cycles long). For a given bin, if the spectral amplitude rose above a *z*-score of 2 for at least three consecutive wave-cycles, then it was considered a ‘1’; otherwise, it was considered a ‘0’. The binary time series was fed through the transfer-entropy algorithm to quantify EC between sites.^69-71^ All connections (efferent-emanating and afferent-receiving) for a given channel were averaged to get a single value for analysis. Transfer-entropy-based EC was computed as a global measure, considering connections to all other channels, for a given site. Supplementary analysis probed possible differences between efferent and afferent.

### Anatomical localization of electrode sites

**Figure 2:**
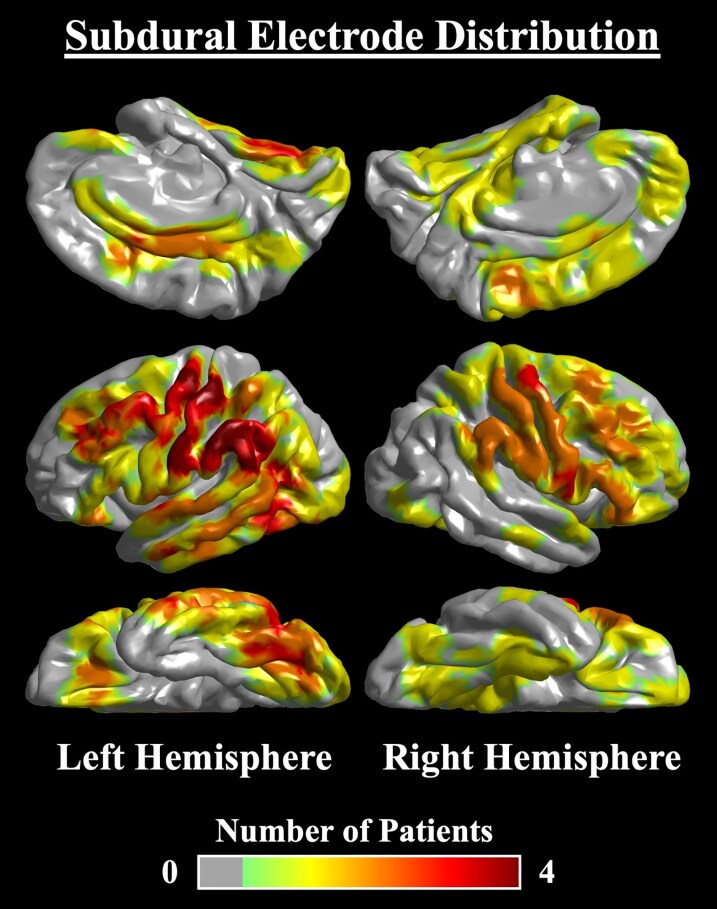
**Subdural electrode distribution.** Anatomical distribution of normative subdural electrodes pooled from the eight two-stage patients who underwent intraoperative ECoG recording at all sevoflurane stages, as well as extraoperative intracranial electroencephalography recording during SWS (*n* = 431 normative electrodes). The heatmap represents the number of patients implanted for a given brain region.

Anatomical localization of electrode sites was performed, as previously done.^62,69,77,78^ Board-certified neurosurgeons used peri-operative photographs to interpolate each patient’s electrodes onto their 3D-reconstructed MRI brain image. FreeSurfer software (https://surfer.nmr.mgh.harvard.edu/) spatially normalized electrodes onto the FreeSurfer average cortical template.^66,82,83^ This allowed group-level anatomical visualization of ECoG/iEEG biomarkers. Although the mathematical mixed models utilized data from all 19 patients (i.e. one- and two-stage patients), the cortical biomarker maps were derived from the eight two-stage patients who received ECoG recording under every anaesthetic stage, as well as iEEG during SWS. Figure 2 shows the anatomical distribution of subdural electrodes from the subset of eight patients (*n* = 431 normative electrodes). Biomarker values from the three individual 1-min recording segments, at a given anaesthetic stage, were averaged to produce one cortical map per condition.

### Dynamic tractography

Dynamic tractography was computed, as done previously, to better understand the cortico-cortical white matter pathways that facilitate propagation of sevoflurane-activated spike-and-wave discharges, approximated by delta–HFO PAC, as well as HFO.^66,69,84-87^ For each anaesthetic stage, DSI Studio software (https://dsi-studio.labsolver.org/) visualized DWI white matter tracts directly connecting cortical sites showing significant co-augmentation (i.e. at least 3 SD above the SWS mean) of the ECoG/iEEG biomarkers. Electrode positions were converted to regions on the Lausanne Brain Atlas^88^ and overlaid on the Human Connectome Project (HCP) whole-brain, white matter template.^89^ The template was made using whole-brain seeding with the following parameters: tracking threshold of 0.7, angular threshold of 70° and step size of 0.3 mm. The open-source HCP dataset includes averaged diffusion MRI scans from ∼1000 participants. The Siemens scanning protocol was performed, as previously published.^89^

### Statistical analysis

#### Relationship between anaesthetic stage and ECoG biomarkers

A linear mixed model determined the normative biomarker response to increasing sevoflurane concentration. Electrodes from all individual patients were pooled for analysis. The dependent variable was the biomarker value. Fixed-effect predictors included: (i) age; (ii) biological sex; (iii) sampled hemisphere; (iv) presence of an MRI lesion; (v) presence of daily seizures; (vi) number of oral anti-seizure medications taken immediately prior to the ECoG recording; (vii) one- versus two-stage surgery; and (viii) anaesthetic stage. Random-effect predictors included patient and intercept. A fixed-effect predictor greater than zero suggests that the ECoG biomarkers increased as a function of sevoflurane concentration, and vice versa. This analysis was repeated for three conditions (isoflurane versus SWS, isoflurane versus sevoflurane and SWS versus sevoflurane) and two electrophysiology biomarkers (delta–HFO PAC and HFO EC). Thus, we employed a Bonferroni correction for six comparisons, taking a two-sided, corrected *P*-value of <0.05 as significant.

#### Fidelity of sevoflurane-activated ECoG biomarkers for intraoperative characterization of epileptogenicity

A binary logistic mixed model was used to determine whether relatively more augmentation of the ECoG/iEEG biomarkers could characterize the epileptogenic status of electrode sites. Electrodes from all individual patients were pooled for analysis. The dependent variable was epileptogenic status (yes/no). Fixed-effect predictors included: (i) age; (ii) biological sex; (iii) sampled hemisphere; (iv) presence of MRI lesion; (v) presence of daily seizures; (vi) number of anti-seizure medications; (vii) one- versus two-stage surgery; and (viii) biomarker value. Random-effect predictors included patient and intercept. An odds ratio greater than one suggests that higher sevoflurane-activated ECoG/iEEG biomarkers were associated with an increased likelihood of characterizing a given site as epileptogenic. This analysis was repeated for the data *z*-scored via both the isoflurane and SWS controls, 2 electrophysiological epilepsy biomarkers (delta–HFO PAC and HFO EC) and 10 anaesthetic stages. Thus, we employed a Bonferroni correction for 40 comparisons, taking a 2-sided corrected *P*-value of <0.05 as significant. The effect size of anaesthetic influence on biomarker values between epileptogenic and non-epileptogenic sites was calculated using Cohen’s *d*, as done previously.^69^ For each patient and each 1-min segment of a given anaesthetic stage, the Cohen’s *d* was calculated as the absolute value of: [(the average biomarker value in epileptogenic sites) minus (the average biomarker value in non-epileptogenic sites)] divided by (the standard deviation of all sites). Values for all patients and all 1-min segments of a given anaesthetic stage were averaged to produce one effect size for each anaesthetic stage. The same calculation was also performed for SOZ versus non-SOZ sites.

#### Seizure outcome classification

Supplementary analysis included both 19 patients who achieved ILAE-defined seizure freedom and 4 patients who did not achieve seizure control following surgery. The same binary logistic mixed model was used to determine whether the subtraction biomarker value could characterize seizure freedom. The subtraction biomarker value for each patient was defined as the average of all resected sites minus the average of all retained sites, as done previously.^62^ This effectively quantified the completeness of resecting delta–HFO PAC and HFO EC sites. Higher subtraction values reflect more complete resection.

## Results

### Normative sevoflurane distribution of delta–HFO PAC

**Figure 3:**
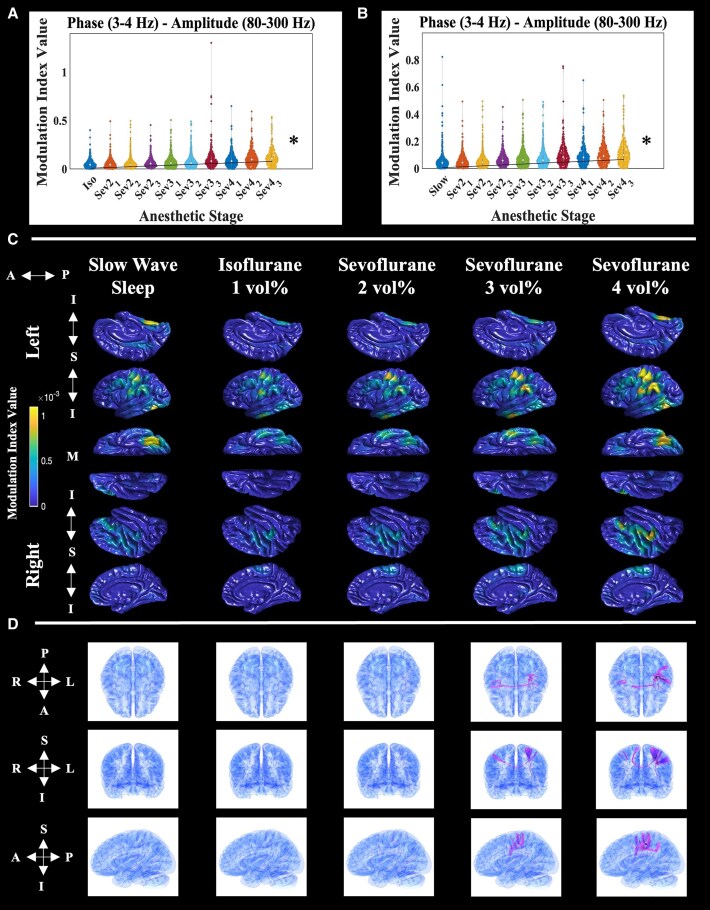
**Normative sevoflurane distribution of delta and HFO phase–amplitude coupling.** Mathematical distribution of ECoG/iEEG-defined delta (3–4 Hz) and HFO (80–300 Hz) phase–amplitude coupling (delta–HFO PAC)—rated by modulation index—from all pooled normative electrode sites, at each anaesthetic stage. Each data point represents a biomarker value measured at a single electrode, in a given anaesthetic stage. The left panel (**A**) shows delta–HFO PAC data from one- and two-stage patients (iso; *n* = 963 normative electrode sites) and a linear mixed model that uses the isoflurane period as the reference. The right panel (**B**) shows delta–HFO PAC data from two-stage patients (slow; *n* = 816 normative electrode sites) and a linear mixed model that uses the SWS period as the reference. In addition, ‘Sev2_1’ denotes the first minute of sevoflurane at a concentration of 2 vol%, and so on. The black trend line anchored at the origin depicts the relationship between delta–HFO PAC and anaesthetic stage, as determined by linear mixed-model analysis. The asterisks represent a significant effect (Bonferroni-corrected *P* < 0.05) of anaesthetic stage on biomarker value, via linear mixed-model analysis. For the linear mixed models, fixed-effect predictors included: (i) age; (ii) biological sex; (iii) sampled hemisphere; (iv) presence of MRI lesion; (v) presence of daily seizures; (vi) number of oral anti-seizure medications taken immediately prior to the ECoG recording; (vii) one- versus two-stage surgery; and (viii) anaesthetic stage. Random-effect predictors included patient and intercept. The dependent variable was the biomarker value. (**C**) Anatomical distribution of normative delta–HFO PAC interpolated onto the FreeSurfer average cortical surface. The data represent normative electrode sites pooled from the eight patients who underwent both extraoperative iEEG recording during SWS, as well as intraoperative ECoG at every anaesthetic stage (*n* = 431 normative electrode sites). Each column represents a different anaesthetic stage. The left and right hemispheres are grouped in the first and last three rows, respectively. Within each hemisphere group, single rows represent different cortical surfaces. For the left hemisphere: *top* row–sagittal–medial, *middle* row–sagittal–lateral and *bottom* row–axial–inferior. The orientation row order is opposite for the right hemisphere. The anterior–posterior (‘A ←→ P’) orientation is listed for all views. In addition, the inferior and superior orientations for the sagittal images are depicted (‘I ←→S ←→ I’), as well as the medial (‘M’) marker for the inferior-axial view. Hotter colours represent sites with relatively higher ECoG biomarker values and vice versa. The spatial coverage of intracranial electrodes is presented in Fig. 2. In areas without intracranial electrode coverage, the absence of biomarker values should be interpreted as a lack of iEEG signal sampling. (**D**) Dynamic tractography depicts DWI white matter streamlines connecting cortical sites with significantly elevated delta–HFO PAC (i.e. >3 SD above the SWS mean). Each column represents a different anaesthetic stage, and each row, from *top* to *bottom*, shows a different brain view: axial, coronal and sagittal, respectively. The orientation for each view is included to the left of the corresponding row. A, anterior; I, inferior; Iso, isoflurane control period; L, left; M, medial; P, posterior; R, right; S, superior; Slow, SWS control period.

We first assessed the normative response of interictal, intraoperative delta–HFO PAC (rated by modulation index) to stepwise increasing sevoflurane. For the isoflurane control, linear mixed-model analysis demonstrated that normative delta–HFO PAC values increased as a function of sevoflurane concentration (Fig. 3A; fixed-effect estimate = 0.007; *t*-value = 18.566; uncorrected *P*-value = 2.24 × 10^−74^; df = 4825). The same trend was seen in the SWS control (Fig. 3B; fixed-effect estimate = 0.006; *t*-value = 16.407; uncorrected *P*-value = 8.64×10^−59^; df = 4478). Linear mixed-model fitting performance was assessed via Q–Q plots of residuals for both controls (Supplementary Fig. 1). A similar calculation did not show a difference between normative isoflurane (1 vol%) and SWS (fixed-effect estimate = −0.002; *t*-value = −0.670; uncorrected *P*-value = 0.503; df = 1240). Figure 3C visualizes the cortical surface distribution of delta–HFO PAC at each anaesthetic stage, for eight patients who received extraoperative iEEG recording plus every intraoperative stage (*n* = 431 normative sites). Prominent posterior delta–HFO PAC enhancement during the SWS condition shifted to sensorimotor prominence by sevoflurane (4 vol%). Dynamic tractography suggested that the significant sevoflurane-induced delta–HFO PAC co-augmentation between Rolandic areas was supported by inter-hemispheric callosal fibres (Fig. 3D).

### Normative sevoflurane distribution of HFO effective connectivity

**Figure 4:**
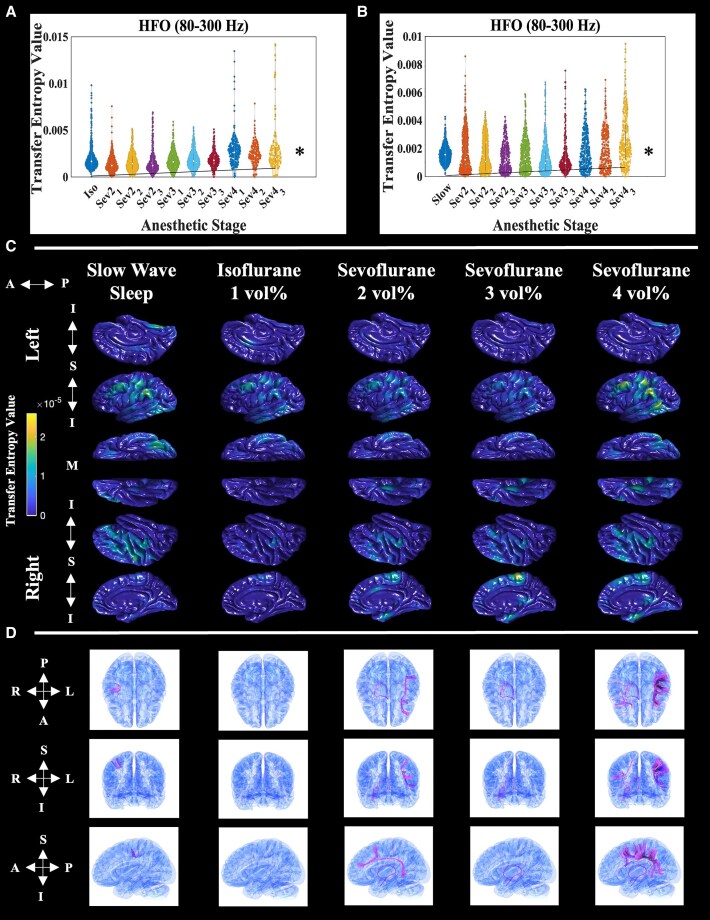
**Normative sevoflurane distribution of HFO EC.** Mathematical distribution of HFO (80–300 Hz) EC—rated by transfer entropy—from all pooled normative electrode sites, at each anaesthetic stage. Each data point represents a biomarker value measured at a single electrode, in a given anaesthetic stage. The left panel (**A**) shows HFO EC data from one- and two-stage patients derived from a spectral amplitude time series *z*-scored in relation to the mean and standard deviation of the isoflurane control (iso; *n* = 963 normative electrode sites) and a linear mixed model that uses the isoflurane period as the reference. The right panel (**B**) shows HFO EC data from two-stage patients derived from a spectral amplitude time series *z*-scored in relation to the mean and standard deviation of the SWS control (slow; *n* = 816 normative electrode sites) and a linear mixed model that uses the SWS period as the reference. In addition, ‘Sev2_1’ denotes the first minute of sevoflurane at a concentration of 2 vol%, and so on. The black trend line anchored at the origin depicts the relationship between HFO EC and anaesthetic stage, as determined by linear mixed-model analysis. The asterisks represent a significant effect (Bonferroni-corrected *P*-values < 0.05) of anaesthetic stage on biomarker value, via linear mixed-model analysis. For the linear mixed models, fixed-effect predictors included: (i) age; (ii) biological sex; (iii) sampled hemisphere; (iv) presence of MRI lesion; (v) presence of daily seizures; (vi) number of oral anti-seizure medications taken immediately prior to the ECoG recording; (vii) one- versus two-stage surgery; and (viii) anaesthetic stage. Random-effect predictors included patient and intercept. The dependent variable was the biomarker value. (**C**) Anatomical distribution of normative HFO EC interpolated onto the FreeSurfer average cortical surface. The data represent normative electrode sites pooled from the eight patients who underwent both extraoperative iEEG recording during SWS, as well as intraoperative ECoG at every anaesthetic stage (*n* = 431 normative electrode sites). Each column represents a different anaesthetic stage. The left and right hemispheres are grouped in the first and last three rows, respectively. Within each hemisphere group, single rows represent different cortical surfaces. For the left hemisphere: *top* row–sagittal–medial, *middle* row–sagittal–lateral and *bottom* row–axial–inferior. The orientation row order is opposite for the right hemisphere. The anterior–posterior (‘A ←→ P’) orientation is listed for all views. In addition, the inferior and superior orientations for the sagittal images are depicted (‘I ←→S ←→ I’), as well as the medial (‘M’) marker for the inferior-axial view. Hotter colours represent sites with relatively higher biomarker values and vice versa. The spatial coverage of intracranial electrodes is presented in Fig. 2. In areas without intracranial electrode coverage, the absence of biomarker values should be interpreted as a lack of iEEG signal sampling. (**D**) Dynamic tractography depicts DWI white matter streamlines connecting cortical sites with significantly elevated HFO EC (i.e. >3 SD above the SWS mean). Each column represents a different anaesthetic stage, and each row, from *top* to *bottom*, shows a different brain view: axial, coronal and sagittal, respectively. The orientation for each view is included to the left of the corresponding row. A, anterior; I, inferior; Iso, isoflurane control period; L, left; M, medial; P, posterior; R, right; S, superior; Slow, SWS control period.

We next determined the normative response of interictal HFO EC (rated by transfer entropy) to stepwise increasing sevoflurane. Linear mixed-model analysis suggested that HFO EC increased as a function of sevoflurane concentration, for both controls (Fig. 4A; isoflurane control: fixed-effect estimate = 6.076×10^−5^; *t*-value = 8.248; uncorrected *P*-value = 2.15 × 10^−16^; df = 4008 | Fig. 4B; SWS control: fixed-effect estimate = 4.107 × 10^−5^; *t*-value = 6.806; uncorrected *P*-value = 1.14 × 10^−11^; df = 4419). Linear mixed-model fitting performance was assessed via Q–Q plots of residuals for both controls (Supplementary Fig. 2). Separating afferent and efferent transfer entropy suggested that they behaved the same as combined (Supplementary Table 2). There was no significant difference between normative isoflurane (1 vol%) and SWS (fixed-effect estimate = 8.207 × 10^−5^; *t*-value = 1.199; uncorrected *P*-value = 0.231; df = 1239). Interpolation of normative sites (*n* = 431 electrodes from eight patients who received extraoperative iEEG recording plus every intraoperative stage) onto an average cortical surface qualitatively showed sevoflurane-activated HFO EC in frontal and parietal areas (Fig. 4C). Dynamic tractography traced prominent intra-hemispheric connections between significant sevoflurane-activated cortical HFO EC foci in frontal and parietal association regions, via the superior longitudinal fasciculus (Fig. 4D).

### Normative sevoflurane distribution of delta effective connectivity

We created a supplementary normative map of interictal, sevoflurane-activated delta EC (rated by transfer entropy). Linear mixed-model analysis showed that sevoflurane increased delta EC, regardless of control (Supplementary Fig. 3A and B and Supplementary Table 3; significance with Bonferroni-corrected *P*-values < 0.05). Linear mixed-model fitting performance was assessed via Q–Q plots of residuals for both controls (Supplementary Fig. 4). Combined, afferent and efferent delta transfer entropy all responded to sevoflurane in the same manner (Supplementary Table 3; significance with Bonferroni-corrected *P*-values < 0.05). Compared to SWS, isoflurane increased delta EC (Supplementary Table 3; significance with Bonferroni-corrected *P*-values < 0.05). Normative sites (*n* = 431 electrodes from eight patients who received extraoperative iEEG recording plus every intraoperative stage), pooled on the FreeSurfer average cortical surface, qualitatively showed prominent delta EC activations in frontal and parietal association areas, along with smaller foci in the sensorimotor strips (Supplementary Fig. 3C). Dynamic tractography suggested that the significant normative delta EC hotspots were connected via both inter-hemispheric callosal fibres and intra-hemispheric superior longitudinal fasciculi (Supplementary Fig. 3D).

### Characterizing epileptogenicity via intraoperative ECoG biomarkers

**Figure 5:**
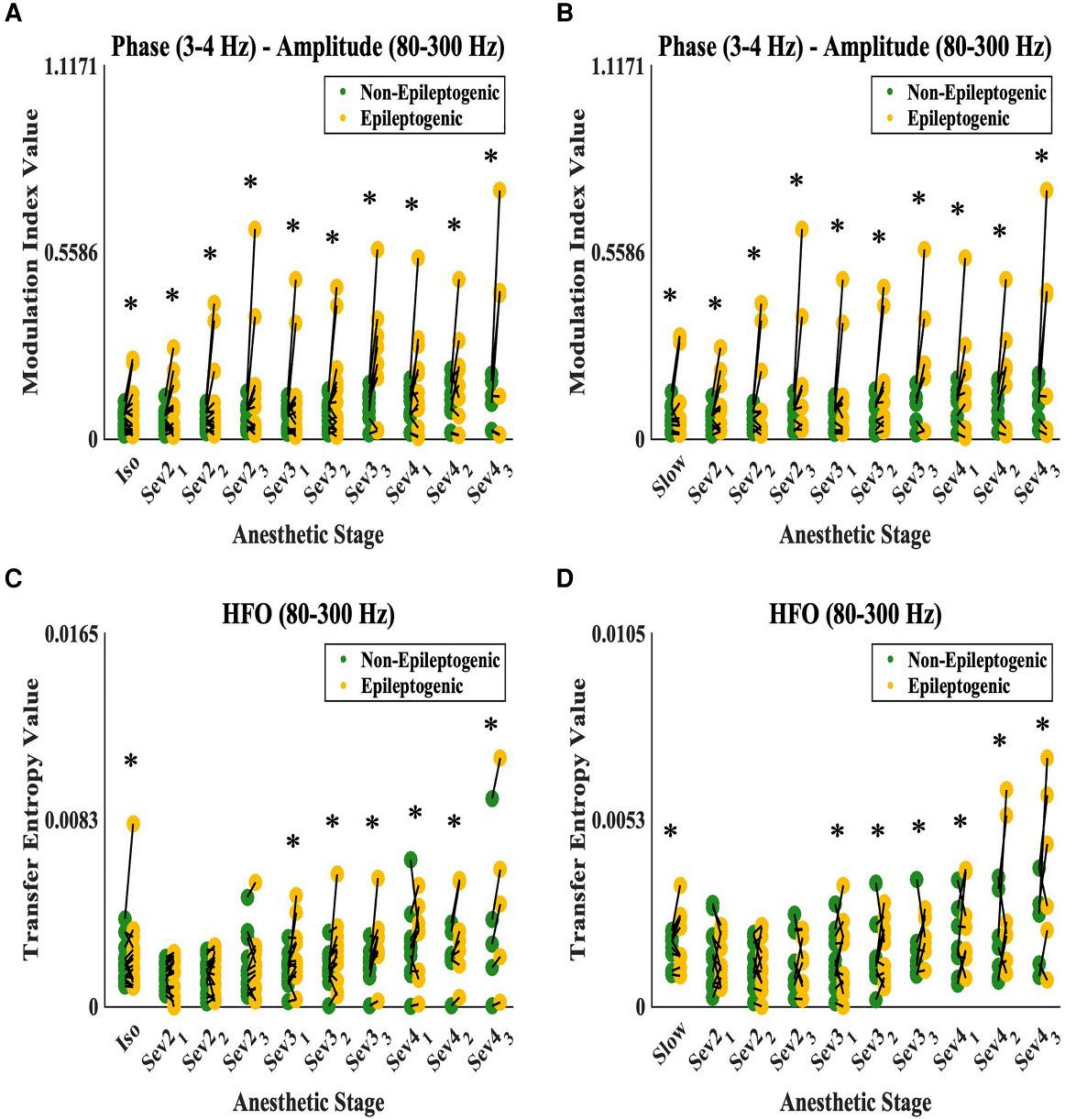
**Sevoflurane-activated ECoG biomarkers characterize epileptogenicity.** Delta (3–4 Hz) and HFO (80–300 Hz) phase–amplitude coupling (delta–HFO PAC rated by modulation index), values from one- and two-stage patients in the (**A**) isoflurane control distribution (*n* = 1608 total electrode sites) and from two-stage patients in the (**B**) SWS control distribution (*n* = 1344 total electrode sites), at each anaesthetic stage. For a given patient, the green and yellow dots represent the average value of all non-epileptogenic and epileptogenic sites, respectively. In each anaesthetic stage, the black lines connect a pair of green-yellow dots from the same patient. The asterisks denote binary logistic mixed-model significance (Bonferroni-corrected *P* < 0.05) for characterizing epileptogenicity via iEEG/ECoG biomarker levels. For the binary logistic mixed models, the dependent variable was the epileptogenic status of electrodes (yes/no). Fixed-effect predictors included: (i) age; (ii) biological sex; (iii) sampled hemisphere; (iv) presence of MRI lesion; (v) presence of daily seizures; (vi) number of anti-seizure medications; (vii) one- versus two-stage surgery; and (viii) biomarker value. Random-effect predictors included patient and intercept. On the *x*-axis, ‘Sev2_1’ denotes the first minute of sevoflurane at a concentration of 2 vol%, and so on. The same analysis is repeated for HFO EC (rated by transfer entropy). Panel (**C**) shows HFO EC values from one- and two-stage patients derived from a spectral amplitude time series *z*-scored against the mean and standard deviation of the isoflurane control (*n* = 1608 total electrode sites). Panel (**D**) shows HFO EC values from two-stage patients derived from a spectral amplitude time series *z*-scored against the mean and standard deviation of the SWS control (*n* = 1344 total electrode sites). ‘Iso’ refers to the isoflurane control period, while ‘Slow’ refers to the SWS control period.

In the next experiment, we pooled all electrode sites, regardless of normative status. Binary logistic mixed-model analysis suggested that relatively higher intraoperative delta–HFO PAC values were associated with an increased likelihood of characterizing a given site as epileptogenic, at every anaesthetic stage (Fig. 5A and B and Table 2). A double dissociation was found between HFO and delta EC values. Compared to non-epileptogenic sites, greater HFO EC (Fig. 5C and D and Table 3) and lower delta EC (Supplementary Fig. 5 and Supplementary Table 4) were associated with epileptogenic electrode sites at sevoflurane (3–4 vol%) and sevoflurane (2–4 vol%), respectively. Greater delta–HFO PAC and HFO EC in epileptogenic sites were also noted during isoflurane (1 vol%) and SWS stages. However, Cohen’s *d* calculation (Supplementary Table 5) showed that this effect was higher during sevoflurane (3–4 vol%). Since epileptogenicity cannot be determined until the seizure outcome is known, we also conducted a parallel supplementary analysis characterizing the SOZ, which is estimated prior to resection surgery. The binary logistic mixed model and Cohen’s *d* analysis showed the same relationship, especially at sevoflurane (3–4 vol%): higher delta–HFO PAC and HFO EC but lower delta EC augmentation characterized SOZ sites (Supplementary Fig. 6 and Supplementary Tables 6–9).

**Table 2:** Binary logistic mixed-model characterization of epileptogenicity based on the delta (3–4 Hz) and HFO (80–300 Hz) phase–amplitude coupling response to anaesthesia

Fixed-effect variable^a^	Number of patients included in the analysis	DF	*t*-Value	Uncorrected *P*-value	Bonferroni-corrected *P*-value^b^	OR
Delta–HFO PAC during isoflurane (1 vol%)	18: One-stage and two-stage	994	3.321	0.000931	0.037	49.413
Delta–HFO PAC during SWS	11: Two-stage only	1279	6.908	7.71 × 10^−12^	3.08 × 10^−10^	183.095
Delta–HFO PAC during the first minute of sevoflurane (2 vol%)	18: One-stage and two-stage	1046	5.951	3.64 × 10^−9^	1.46 × 10^−7^	313.575
	11: Two-stage only	959	5.995	2.88 × 10^−9^	1.15 × 10^−7^	338.044
Delta–HFO PAC during the second minute of sevoflurane (2 vol%)	18: One-stage and two-stage	1051	5.457	6.02 × 10^−8^	2.41 × 10^−6^	64.474
	11: Two-stage only	951	5.496	5.00 × 10^−8^	2.00 × 10^−6^	68.855
Delta–HFO PAC during the third minute of sevoflurane (2 vol%)	18: One-stage and two-stage	801	4.871	1.34 × 10^−6^	5.36 × 10^−5^	33.224
	11: Two-stage only	656	4.93	1.04 × 10^−6^	4.16 × 10^−5^	40.351
Delta–HFO PAC during the first minute of sevoflurane (3 vol%)	18: One-stage and two-stage	977	5.03	5.84 × 10^−7^	2.34 × 10^−5^	38.738
	11: Two-stage only	915	5.08	4.58 × 10^−7^	1.83 × 10^−5^	43.241
Delta–HFO PAC during the second minute of sevoflurane (3 vol%)	18: One-stage and two-stage	939	6.318	4.08 × 10^−10^	1.63 × 10^−8^	80.291
	11: Two-stage only	831	6.361	3.30 × 10^−10^	1.32 × 10^−8^	97.417
Delta–HFO PAC during the third minute of sevoflurane (3 vol%)	18: One-stage and two-stage	601	6.46	2.16 × 10^−10^	8.64 × 10^−9^	58.116
	11: Two-stage only	555	6.039	2.84 × 10^−9^	1.14 × 10^−7^	53.66
Delta–HFO PAC during the first minute of sevoflurane (4 vol%)	18: One-stage and two-stage	698	5.561	3.82 × 10^−8^	1.53 × 10^−6^	47.28
	11: Two-stage only	621	5.341	1.30 × 10^−7^	5.2 × 10^−6^	44.682
Delta–HFO PAC during the second minute of sevoflurane (4 vol%)	18: One-stage and two-stage	547	6.119	1.79 × 10^−9^	7.16 × 10^−8^	132.739
	11: Two-stage only	555	6.035	2.91 × 10^−9^	1.16 × 10^−7^	132.745
Delta–HFO PAC during the third minute of sevoflurane (4 vol%)	18: One-stage and two-stage	430	8.078	6.66 × 10^−15^	2.66 × 10^−13^	21.41
	11: Two-stage only	472	6.713	5.49 × 10^−11^	2.20 × 10^−9^	302.272

DF, degrees of freedom; OR, odds ratio.

^a^The delta–HFO PAC value (rated by modulation index), as an ECoG/iEEG biomarker, was incorporated into the multivariate binary logistic mixed-model analysis, which included eight fixed-effect predictors: (i) age; (ii) biological sex; (iii) sampled hemisphere; (iv) presence of an MRI lesion; (v) presence of daily seizures; (vi) number of anti-seizure medications; (vii) one- versus two-stage surgery; and (viii) biomarker value. Patient and intercept were considered random-effect variables. The binary response variable was the epileptogenic- or non-epileptogenic status of electrodes (1 or 0, respectively).

^b^Bonferroni correction was applied due to the 40 analyses conducted, and significant time points are bolded (Bonferroni-corrected *P*-value < 0.05).

**Table 3:** Binary logistic mixed-model characterization of epileptogenicity based on the HFO EC (80–300 Hz) response to anaesthesia

Fixed-effect variable^a^	Number of patients included in the analysis^b^	DF	*t*-Value	Uncorrected *P*-value	Bonferroni-corrected *P*-value^c^	OR
**HFO EC during isoflurane (1 vol%)**	**18: One-stage and two-stage**	**994**	**3**.**551**	**0**.**00040**	**0**.**016**	**1.06×10** ^**135**^
**HFO EC during** SWS	**11: Two-stage only**	**1279**	**7**.**259**	**6.74** **×** **10^−13^**	**2.70** **×** **10^−11^**	**3.50** **×** **10^301^**
HFO EC during the first minute of sevoflurane (2 vol%)	18: One-stage and two-stage	858	1.908	0.057	>0.99	4.63 × 10^104^
	11: Two-stage only	941	−2.256	0.024	0.96	3.58 × 10^−84^
HFO EC during the second minute of sevoflurane (2 vol%)	18: One-stage and two-stage	854	2.372	0.018	0.72	1.19 × 10^132^
	11: Two-stage only	928	−0.55	0.583	>0.99	2.32 × 10^−22^
HFO EC during the third minute of sevoflurane (2 vol%)	18: One-stage and two-stage	652	1.266	0.206	>0.99	1.44 × 10^71^
	11: Two-stage only	643	−0.381	0.703	>0.99	3.08×10^−20^
**HFO EC during the** fir**st minute of sevoflurane (3 vol%)**	**18: One-stage and two-stage**	**815**	**5**.**168**	**2.98** **×** **10^−7^**	**1.19** **×** **10^−5^**	**9.94** **×** **10^207^**
	**11: Two-stage only**	**898**	**3**.**436**	**0.00062**	**0.025**	**1.45** **×** **10^107^**
**HFO EC during the** seco**nd minute of sevoflurane (3 vol%)**	**18: One-stage and two-stage**	**744**	**5**.**904**	**5.38** **×** **10^−9^**	**2.15** **×** **10^−7^**	**^d^**
	**11: Two-stage only**	**816**	**4**.**563**	**5.83** **×** **10^−6^**	**2.33** **×** **10^−4^**	**1.56** **×** **10^174^**
**HFO EC during the** thi**rd minute of sevoflurane (3 vol%)**	**18: One-stage and two-stage**	**449**	**6**.**138**	**1.84** **×** **10^−9^**	**7.36** **×** **10^−8^**	**^d^**
	**11: Two-stage only**	**547**	**6**.**761**	**3.52** **×** **10^−11^**	**1.41** **×** **10^−9^**	**6.19** **×** **10^295^**
**HFO EC during the** fir**st minute of sevoflurane (4 vol%)**	**18: One-stage and two-stage**	**512**	**4**.**99**	**8.31** **×** **10^−7^**	**3.32** **×** **10^−5^**	**4.76** **×** **10^179^**
	**11: Two-stage only**	**604**	**4**.**584**	**5.55** **×** **10^−6^**	**2.22** **×** **10^−4^**	**1.15** **×** **10^153^**
**HFO EC during the** seco**nd minute of sevoflurane (4 vol%)**	**18: One-stage and two-stage**	**411**	**6**.**996**	**1.07** **×** **10^−11^**	**4.28** **×** **10^−10^**	^d^
	**11: Two-stage only**	**538**	**7**.**806**	**3.11** **×** **10^−14^**	**1.24** **×** **10^−12^**	**7.73** **×** **10^277^**
**HFO EC during the** thi**rd minute of sevoflurane (4 vol%)**	**18: One-stage and two-stage**	**315**	**6**.**12**	**2.78** **×** **10^−9^**	**1.11** **×** **10^−7^**	**4.33** **×** **10^273^**
	**11: Two-stage only**	**460**	**8**.**01**	**9.55** **×** **10^−15^**	**3.82** **×** **10^−13^**	**1.72** **×** **10^288^**

DF, degrees of freedom; OR, odds ratio.

^a^The HFO EC value (rated by transfer entropy), as an ECoG/iEEG biomarker, was incorporated into the multivariate binary logistic mixed-model analysis, which included eight fixed-effect predictors: (i) age; (ii) biological sex; (iii) sampled hemisphere; (iv) presence of an MRI lesion; (v) presence of daily seizures; (vi) number of anti-seizure medications; (vii) one- versus two-stage surgery; and (viii) biomarker value. Patient and intercept were considered random-effect variables. The binary response variable was the epileptogenic- or non-epileptogenic status of electrodes (1 or 0, respectively).

^b^In the analysis including both one- and two-stage surgery patients, transfer entropy was calculated using a spectral amplitude time series that was normalized via *z*-scoring based on the mean and standard deviation of the isoflurane (1 vol%) period. In the analysis with only two-stage surgery patients, transfer entropy was calculated using a spectral amplitude time series that was normalized via *z*-scoring based on the mean and standard deviation of the SWS period.

^c^Bonferroni correction was applied due to the 40 analyses conducted, and significant time points are bolded (Bonferroni-corrected *P*-value < 0.05).

^d^The odds ratio was not shown in SPSS due to the enormous effect size.

### Intraoperative prediction of seizure freedom

A final supplementary analysis sought to determine whether the completeness of resecting regions displaying high values of sevoflurane-activated delta–HFO PAC and/or EC could predict postoperative ILAE Class I seizure freedom. For each patient, subtracting the average ECoG/iEEG biomarker value of all retained sites from the average of all resected sites served as a proxy for resection completeness. However, the subtraction values did not significantly predict seizure freedom (Supplementary Tables 10–12).

## Discussion

### Significance and innovation

To our knowledge, this is the first study to depict the normative distribution of ECoG/iEEG-based intraoperative delta–HFO PAC and HFO EC, at stepwise increasing concentrations of sevoflurane anaesthesia. The novelty of this work includes: (i) beginning to illuminate the normative cortical distribution of ECoG/iEEG-based delta–HFO PAC, HFO EC and delta EC, at various sevoflurane, isoflurane and SWS stages; (ii) using dynamic tractography to delineate cortico-cortical white matter pathways potentially facilitating the propagation of sevoflurane-induced delta–HFO PAC and HFO EC across normative areas; and (iii) showing the fidelity of sevoflurane-activated delta–HFO PAC, HFO EC and delta EC for intraoperative characterization of epileptogenic and SOZ sites.

Relatively few studies have investigated the effects of sevoflurane on HFO signalling in patients with focal epilepsy. Previous studies reported that the occurrence rates of spikes and HFO increased most prominently in the SOZ under sevoflurane.^50,51^ The present findings also align with our preliminary investigation, which analysed intraoperative ECoG data from a separate cohort of eight patients with drug-resistant focal epilepsy. Data from both cohorts independently suggest that sevoflurane generally elevates delta–HFO PAC and HFO EC, with the greatest increases observed in epileptogenic sites.^68,69^ Moreover, these studies demonstrated a double dissociation, wherein epileptogenic regions exhibit relatively higher delta–HFO PAC and HFO EC but lower delta EC augmentation under sevoflurane. Slow waves are thought to reflect neural inhibition^90-92^; whereas, HFO coupled with spike-and-wave discharges are attributed to neural excitation.^93,94^ This supports the notion that sites experiencing relatively less inhibitory delta influence concurrently display greater levels of HFO. Another recent study by Uda *et al*. (2024) showed that the highest degree of sevoflurane-enhanced HFO and spike rates were in SOZ sites.^72^ While Uda *et al*. (2024) examined the occurrence rates of HFO events in single channels, the present study analysed HFO coupling with slow waves and network properties, specifically delta–HFO PAC and EC. In addition, the current investigation included dynamic tractography to delineate possible white matter pathways facilitating HFO propagation under sevoflurane. The results of these analyses infer that the ability to characterize epileptogenicity was optimized between sevoflurane 3–4 vol%.^68,69,72,95^

Numerous studies suggest that HFOs serve as a compelling biomarker of epilepsy, with their removal reportedly associated with postoperative seizure freedom.^40-69,96-98^ Clinical investigations have also implicated elevated delta–HFO PAC in identifying seizure foci.^57-59,62-66,69,99-101^ However, it is established that physiological HFO exist in the brain and are thought to play a role in memory consolidation.^73,102-104^ Similarly, physiological phase–amplitude coupling is suggested to integrate distant spatial signals into singular cognitive events.^67,105,106^ When considering these ECoG features as epilepsy biomarkers, distinguishing physiological activity from genuinely pathological signals is crucial. This distinction is particularly relevant given that sevoflurane is known to induce epileptiform EEG signals and seizure-like movements even in non-epileptic patients.^29,30^ Focusing on HFO network dynamics may help address this challenge, as epilepsy is associated with abnormal network connectivity.^69,107-112^ Our results suggest that while sevoflurane enhances HFO EC even in normative sites, the most pronounced augmentation occurs in the epileptogenic and seizure-onset zones. This aligns with studies demonstrating that distinct spike and HFO propagation patterns characterize epileptogenic brain networks.^69,113-115^ Specifically, Tamilia *et al*. (2021) and others have suggested that within pathological brain networks, the onset location of HFO and spike propagation trains is most indicative of the epileptogenic zone.^116-119^ Our dynamic tractography results attempt to provide an anatomical framework for these neural propagations, albeit within normative sites under sevoflurane. Previous clinical iEEG studies have implicated white matter as a conduit for epileptiform signal propagation.^69,85,120-123^ Consequently, these fibres may serve as critical targets for surgical disconnection to prevent pathological discharges from reaching regions responsible for clinical manifestations.

Our novel, normative sevoflurane brain maps provide a preliminary reference to improve distinguishing off-target anaesthetic-related effects from genuine epileptogenic augmentation. While more investigation is needed, delta–HFO PAC (rated by modulation index) and HFO EC (rated by transfer entropy) represent exciting objective intraoperative ECoG epilepsy biomarkers. Using sevoflurane to rapidly and reversibly augment these electrophysiological metrics during surgery could help reduce a severe diagnostic burden by mitigating the need for intracranial electrode implants and extraoperative iEEG recording. This would optimize treatment cost-effectiveness, while maintaining postoperative seizure outcomes comparable with conventional methods. Even in cases where two-stage surgery is still necessary, sevoflurane-based guidance could improve intracranial electrode placement, although future studies are needed to confirm this point.

### Possible mechanisms

While the exact mechanism of how sevoflurane enhances epileptiform HFO signal generation remains unclear, we hypothesize the following working model. It is accepted that sevoflurane potentiates inhibitory GABAergic neurotransmission.^124-127^ This hyperpolarizing drive disinhibits T-type calcium channels in thalamocortical relay neurons, which then enter burst firing mode. Bursting in the thalamus entrains cortical synchronization and, via corticothalamic projections, sets up a feedback resonance with the thalamus.^128-133^ Cortical interneurons organize the thalamocortical bursts into delta waves.^128-133^ However, extensive sevoflurane exposure is known to overwhelm the cellular GABAergic machinery responsible for coordinating cortical activity, and this impaired inhibition leads to asynchronous neuronal firing.^127,134-136^ HFO might then emerge from the combination of malfunctioning perisomatic inhibition plus large waves of sevoflurane-driven bursting activity, leading to irregular summation of asynchronous cortical firing.^91^ Since epileptic tissue is thought to have disrupted inhibition,^137,138^ it would inherently be more sensitive to such effects. This phenomenon might also help explain the double dissociation between higher delta–HFO PAC and HFO EC but lower delta EC, in epileptogenic and SOZ sites. Sevoflurane (3–4 vol%) may have paradoxically blunted delta activity, disinhibiting HFO and spike-and-wave propagation, even in normative brain regions. This model is further supported by the findings that: (i) the GABA_A_ antagonist bicuculline can cause epileptiform discharges; and (ii) penicillin is believed to operate via a similar mechanism to spur EEG spikes.^134,139^ We believe the present study begs basic science questions on how sevoflurane alters neural signalling. A better understanding of the cellular and circuit mechanisms generating HFO and delta waves could provide avenues to improve the system for optimal clinical use.

### Limitations and future directions

There were several limitations to the present work that should be addressed. One criticism is the small number of patients used in this study. Thus, it is possible that the qualitative signal distribution displayed on the brain maps may be partially skewed toward the anatomical distribution of implanted electrodes in these few patients. Ethical concerns constrain invasive sampling only to implicated brain regions. Increasing the number of subjects would certainly improve resolution. However, we were limited by the number of patients who fit the inclusion criteria. The mixed-model approach helps overcome this issue because: (i) it allows each individual electrode site to count toward the *n*-number; and (ii) it allows pooling of electrodes across all patients. Since ‘patient’ was included as a random-effects variable in the mixed models, baseline differences across subjects were inherently accounted for. This allowed us to pool all electrode sites together for the analysis. For example, the isoflurane control period included *n* = 1608 electrode sites; meaning that with a power of 0.8 and an alpha = 0.05, we were able to detect a small effect size of around 0.01. Thus, our models have adequate statistical power to assess the impact of sevoflurane on the ECoG/iEEG biomarkers.

However, our subtraction models applied to 23 patients did not demonstrate that ECoG/iEEG biomarkers could distinguish between patients who achieved postoperative seizure freedom and those who did not. We computed subtraction as the difference between the average of all retained and resected sites, treating this measure as an estimate of the completeness of biomarker site resection. A previous study from our group involving over 100 patients showed that subtraction biomarker values during SWS classified patients with ILAE Class I seizure outcomes.^62^ An alternative analytic approach may better quantify the completeness of resecting high-value biomarker sites. Future studies may benefit from normalizing subtraction values, as performed in a study of 109 patients,^55^ to mitigate baseline differences across patients before statistical analysis. The failure to demonstrate the predictive utility of ECoG/iEEG biomarkers may be attributed to the small sample size. The present analysis included only 4 poor-outcome patients and 19 ILAE Class I seizure-free patients. With a sample size of 23 and *α* = 0.05, statistical power was only 0.32 to detect a large effect size (0.8). Increasing the sample size to 80 would be necessary to achieve sufficient power to detect this effect. Another criticism involves pooling one- and two-stage surgery patients for analysis. We accounted for this potential confounder in our statistical models, which failed to find any significant effect of surgical staging on the outcomes. Also, both groups received identical intraoperative ECoG recording paradigms in the presence of stepwise increasing sevoflurane. The same may be said for pooling patients aged 4–18 years because the brain undergoes dramatic development during that time. The anatomical distribution of physiological delta–HFO PAC shifts as children grow.^66^ The best way to account for these dynamic cerebral changes would be to stratify our analysis based on patient age. However, we did not have the appropriate sample size to conduct such an analysis. We, instead, incorporated age as a fixed-effect factor in our mixed-model statistics, as done previously.^62,68,69^ Our analysis in this study likewise failed to find a significant effect of age on outcomes. At least within the bounds of the studies reported here, treatment plan and age did not affect outcome.

Volume conduction is another potential issue impacting the interpretation of delta–HFO PAC and EC. Mercier *et al*. (2022) published guidelines and good practices for the analysis of human iEEG.^140^ They suggest that local bipolar referencing is the optimal referencing scheme for ECoG/iEEG-derived HFO. This technique mitigates undesired effects such as volume conduction and other artefacts. We employed bipolar referencing in the current study to account for such effects, but it must be considered that this technique does not fully eliminate these factors. Finally, not all electrodes were uniformly included for each anaesthetic stage due to either electrographic artefacts and/or, in limited cases, physiological factors prompting the anesthesiologist to cease increasing sevoflurane. The mixed-model approach sufficiently handled this missing data and allowed us to account for biological variability among patients. An additional criticism is that the Q–Q plots suggest the data may deviate from linearity. To mitigate the non-Gaussian distribution often observed in electrophysiological data, log-transformation has been commonly applied.^141-145^ Hence, we conducted a supplementary analysis using log-transformed biomarker data to compute both linear mixed models and binary logistic mixed models. The log-transformed results generally recapitulated the main results of the study (Supplementary Figs 1, 2 and 4; Supplementary Tables 13–20). The replicated relationship in a nonlinear model supports the possibility that the association between anaesthesia and iEEG measures is partially nonlinear. These significant findings—observed across both linear and nonlinear models—are unlikely to be attributable to noise from model overfitting alone.

In the future, we plan to consider prospective studies to: (i) definitively assess whether adding sevoflurane-guidance to intracranial electrode implantation leads to better postoperative seizure control; and (ii) to determine whether bolstering standard spike-based visualization with delta–HFO PAC and HFO EC can improve the chances of postoperative seizure freedom.

## Supplementary Material

fcaf403_Supplementary_Data

## Data Availability

All data used in this study are available upon reasonable request to the corresponding author. The transfer entropy code is available at GitHub: https://github.com/efire11/Transfer-Entropy.git.
